# Dr. Hiram Corson

**Published:** 1896-04

**Authors:** 


### Obituary.

Dr. Hiram Corson, of Plymouth Meeting, Pa., died at his residence March 4, 1896, at the advanced age of 92 years. He was said to be the oldest physician engaged in practice in the United States, and a remarkable fact connected with his history is, that he was born at Plymouth Meeting where he lived and practised during his entire lifetime. In speaking of his death, the *Lehigh Valley Medical Magazine* says that Dr. Corson was a man of skill, of probity, a defender of principle, a sustainer of all true associated effort for the profession of medicine.

Dr. Corson was an honorary member of the Harrisburg (Pa.) Pathological Society; associate member of the Philadelphia Obstetrical Society; associate fellow of the College of Physicians of Philadelphia, and permanent member (president, 1852,) of the Pennsylvania Medical Society and of the American Medical Association, and an honorary fellow of the American Association of Obstetricians and Gynecologists.

